# A clustering method for single-cell RNA sequencing data based on denoising and masking learning

**DOI:** 10.3389/fbinf.2026.1758257

**Published:** 2026-03-03

**Authors:** Shuang Xu, Wen Yan, Bin Zhang, Hong Qi, Kai Wang

**Affiliations:** 1 Department of Anesthesiology, The Second Hospital of Jilin University, Changchun, China; 2 College of Computer Science and Technology, Jilin University, Changchun, China

**Keywords:** cell clustering, denoising autoencoder, masked autoencoder, single-cell RNA sequencing, zero-inflated negative binomial (ZINB)

## Abstract

**Introduction:**

Single-cell RNA sequencing (scRNA-seq) enables high-throughput analysis of gene expression at single-cell resolution and plays a crucial role in studying cellular heterogeneity, tissue development, and disease mechanisms. However, scRNA-seq data are characterized by high dimensionality, sparsity, technical noise, and prevalent dropout events, which pose substantial challenges to conventional clustering approaches.

**Methods:**

To address these challenges, we propose scDMAC, a novel clustering framework for single-cell RNA sequencing data based on denoising and masking learning. The method integrates a zero-inflated negative binomial (ZINB)-based denoising autoencoder with a masking autoencoder. First, the ZINB-based autoencoder models count distribution and dropout events to denoise gene expression data. Subsequently, a tailored masking strategy is applied to the denoised data to learn gene-wise correlations through reconstruction.

**Results:**

Extensive experiments conducted on multiple benchmark scRNA-seq datasets demonstrate that scDMAC achieves superior clustering accuracy and stability compared with state-of-the-art methods. The proposed framework consistently improves clustering performance across diverse datasets, highlighting its robustness to noise and sparsity.

**Discussion:**

By effectively combining probabilistic denoising with masking-based representation learning, scDMAC provides a powerful solution for addressing dropout and sparsity issues in scRNA-seq data. The improved clustering performance suggests that integrating distribution-aware denoising with feature reconstruction enhances the extraction of biologically meaningful representations, making scDMAC a promising tool for single-cell transcriptomic analysis.

## 1 Introduction

Recent advances in high-throughput sequencing, single-cell isolation, and bioinformatics have enabled multimodal, single-cell-level interrogation of biological systems (Wu et al., 2014; Hong et al., 2020; He et al., 2025). Among single-cell technologies, single-cell RNA sequencing (scRNA-seq) stands out for its ability to resolve transcriptional states at cellular resolution, permitting precise identification of cell types and subpopulations, reconstruction of developmental trajectories, and dissection of molecular mechanisms underlying health and disease (Lopez et al., 2018; Ranjan et al., 2021). As single-cell studies scale across tissues, conditions, and laboratories, robust computational methods (Kinker et al., 2020; Flores et al., 2022) that can extract meaningful biological signals from noisy, sparse measurements are increasingly essential for biological discovery and translational applications such as biomarker identification and therapeutic target prioritization (Haque et al., 2017; Su et al., 2022).

Clustering remains a foundational step in scRNA-seq analysis, but it is challenged by properties inherent to these data: extreme sparsity (many zero or near-zero counts), high dimensionality (tens of thousands of genes per cell), andmixed sources of variation, including true biological heterogeneity and technical noise (dropout events, varying capture efficiency, and limited sequencing depth) (Conesa et al., 2016; Qi et al., 2020; Zhao et al., 2021; Ghorbani et al., 2024; Gong et al., 2018). Sparsity and high dimensionality exacerbate the curse of dimensionality, reducing the discriminative power of conventional distance metrics and degrading the performance of classical clustering methods. Moreover, zero inflation and measurement noise obscure subtle but biologically important gene–gene relationships that are critical for accurate cell-type separation and downstream interpretation (Stegle et al., 2015; Camara, 2018).

Existing ZINB-based models primarily focus on modeling count distributions and dropout events, but often overlook explicit modeling of gene–gene dependencies. Conversely, recent deep clustering methods emphasize representation learning but typically rely on generic reconstruction objectives that are insensitive to biological sparsity patterns. As a result, existing approaches struggle to simultaneously address technical noise, zero inflation, and contextual gene relationships within a unified framework.

To address these challenges, we introduce scDMAC, a unified framework that couples principled probabilistic denoising with contextual masked reconstruction to produce compact, biologically informative embeddings for clustering. Specifically, scDMAC differs fundamentally from existing composite methods such as scDeepCluster and scziDesk in three aspects:

1. The ZINB-based denoising module in scDMAC is used as an explicit pre-denoising stage rather than being jointly optimized with clustering, which stabilizes subsequent representation learning;
2. A gene-wise masked autoencoder is introduced after denoising to explicitly model inter-gene dependencies via contextual reconstruction, which is absent in prior ZINB-based clustering frameworks;
3. scDMAC incorporates an adaptive mask prediction and weighted reconstruction strategy, enabling the model to focus learning capacity on corrupted genes while preserving biological signal, rather than treating all reconstruction errors equally.

Together, these design choices allow scDMAC to address both zero inflation and gene dependency learning in a coordinated manner, going beyond architectural variations of existing deep clustering approaches. We evaluate scDMAC on multiple widely used scRNA-seq benchmarks and demonstrate consistent improvements in clustering accuracy, stability, and robustness to dropout compared with state-of-the-art methods. scDMAC delivers clearer separation of canonical cell types, more reliable identification of rare populations, and improved reproducibility across noisy conditions. Collectively, these results show that combining a statistically grounded noise model with masked contextual learning is an effective strategy for extracting biologically meaningful embeddings from scRNA-seq data, thereby improving downstream tasks such as cell-type annotation, trajectory inference, and differential expression analysis.

## 2 Methods

**FIGURE 1:**
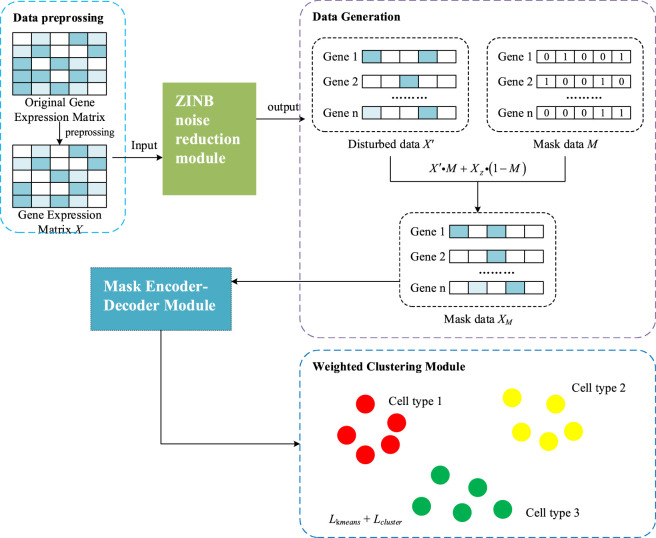
The flowchart of single-cell RNA sequencing data based on denoising and masking learning (scDMAC).

Figure 1 shows the flowchart of the single-cell RNA sequencing cluster method based on denoising and masking learning. To optimize single-cell RNA sequencing (scRNA-seq) data for contrastive learning models, the data first undergoes normalization and log transformation, followed by gene filtering, and finally construction of a k-nearest neighbor (KNN) graph based on cosine distance.

scRNA-seq data often exhibits substantial variation in total gene expression per cell due to differences in sequencing depth, which compromises the comparability of expression values across cells. To mitigate this technical bias, expression values are normalized. Specifically, for the expression value *X_ij_* of gene *j* in cell *i*, the normalized value is calculated in Equation 1.

$$X_{ij}^{norm}=\frac{X_{ij}}{\sum_{j=1}^{G}X_{ij}}\cdot s_{0}$$ (1)

where *s*_0_ is a scaling factor (set to 10,000 in this study). This adjusts the total expression of each cell to a common scale, reducing the impact of sequencing depth.

Log1p normalization, which is defined as Equation 2, is applied after denoising and is only used for downstream masked representation learning and clustering. This separation ensures both statistical validity of the ZINB likelihood and numerical stability for deep representation learning.

$$X_{ij}^{\log}=\ln\left(X_{ij}^{norm}+1\right)$$ (2)

This helps approximate a normal distribution, making the data more suitable for contrastive learning. Subsequently, highly variable genes are selected using the Scanpy package to minimize the influence of uninformative features. The resulting preprocessed matrix *X* serves as input to the model. Finally, a KNN graph is constructed using cosine distance to represent cell neighborhoods.

At the same time, this study uses data augmentation to enhance model performance by generating variations of the original data. For scRNA-seq data, designed augmentation helps simulate real-world data distribution, improving imputation and downstream tasks such as clustering. This section applies three augmentation strategies:

Masking Gene Expressions: Randomly selected genes (10%) have their expressions set to zero. This mimics “dropout” events common in scRNA-seq data, encouraging the model to learn contextual relationships for predicting masked values.

Adding Gaussian Noise: To simulate technical variability from sequencing or handling, Gaussian noise with a variance of 0.6 is added, which improves model robustness to noise, especially useful in datasets with high technical variation.

Swapping Expressions Between Neighboring Cells: Based on the KNN graph, a cell’s expressions are randomly swapped with those of its neighbors at a ratio of 0.2. This promotes local structural variability and reduces over-reliance on fixed neighborhood patterns.

**FIGURE 2:**
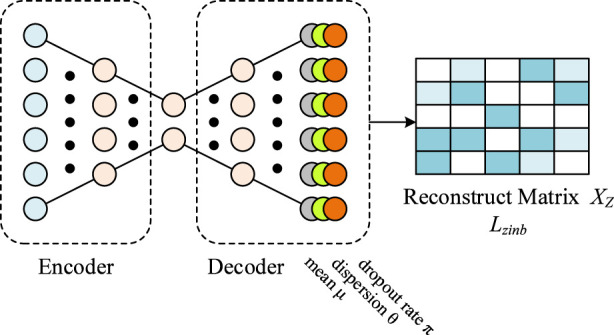
The structure of ZINB noise reduction module.

To denoise the gene expression matrix *X* and capture key characteristics of scRNA-seq data, such as high sparsity and overdispersion. A Zero-Inflated Negative Binomial (ZINB)-based autoencoder is employed, as shown in Figure 2. This model integrates an encoder with a denoising autoencoder architecture inspired by DCA, enhancing its ability to handle scRNA-seq noise and dropout effects. The ZINB module probabilistically models dropout events via the zero-inflation parameter, capturing technical zeros, while the masking strategy serves as a self-supervised regularization mechanism rather than an explicit zero generator. Masked values are only introduced during training and are not interpreted as biological zeros. By decoupling probabilistic dropout modeling from masking-induced perturbations, The preprocessed expression matrix *X* is input into a deep count autoencoder, which uses a ZINB-based loss to reconstruct a denoised expression matrix *X*_*z*_. The ZINB distribution is parameterized by three components: mean (*μ*), dispersion (*θ*), and dropout probability (*π*). The probability of an observed count is defined as Equations 3, 4.

$$\text{NB}\left(X|\mu ,\theta \right)=\frac{\Gamma \left(X+\theta \right)}{X!\Gamma \left(\theta \right)}{\left(\frac{\theta }{\theta +\mu }\right)}^{\theta }{\left(\frac{\mu }{\mu +\theta }\right)}^{X}$$ (3)

$$\text{ZINB}\left(X|\pi ,\mu ,\theta \right)=\pi \delta \left(X\right)+\left(1-\pi \right)\text{NB}\left(X|\mu ,\theta \right)$$ (4)

where Γ denotes the gamma function and δ(*X*)represents a point mass at zero. Unlike standard autoencoders, the ZINB model uses three separate fully connected output layers connected to the decoder’s final hidden layer to estimate the three parameters, as Equations 5–7.

$$\Theta =\exp\left(W_{\theta }D\right)$$ (5)

$$M=\text{diag}\left(s_{i}\right)\times \exp\left(W_{\mu }D\right)$$ (6)

$$\Pi =\text{sigmoid}\left(W_{\pi }D\right)$$ (7)

Here, Θ, *M*, and *Π* denote the matrices of dispersion, mean, and dropout probability, respectively. *W*_*μ*_, *W*_*θ*_ and *W*_*π*_ are the weight matrices for each parameter head, and *D* is the output from the last hidden layer of the decoder. The exponential function ensures non-negativity for mean and dispersion, while sigmoid constrains the dropout probability to [0,1]. The scaling factor *s*_*i*_, obtained during preprocessing, adjusts for cell-specific library size.

The loss function for denoising is the negative log-likelihood of the ZINB distribution as Equation 8.

$$L_{zinb}=-\log\left(\text{ZINB}\left(X|\pi ,\mu ,\theta \right)\right)$$ (8)

Following the denoising step, variability and perturbation are introduced into the denoised gene expression matrix *X*_*z*_ through the following procedure:

First, the expression values of each gene are randomly shuffled within the matrix to preserve intra-gene correlations, resulting in a perturbed matrix *X'*.

Next, a masking matrix *M* is generated using a Bernoulli distribution *Bernoulli* (*p*_*j*_) for each gene as Equation 9, where *p*_*j*_ controls the masking probability for the *j*th gene:

$$M_{ij}∼\text{Bernoulli}\left(p_{j}\right)$$ (9)

Here, *M*_*ij*_ represents the element in the *i*th row and *j*th column of the mask.

Finally, the masked gene expression matrix *X*_*M*_ is obtained via element-wise operations as Equation 10.

$$X_{M_{ij}}=X_{ij}^{\prime }\cdot M_{ij}+X_{z}\cdot \left(1-M_{ij}\right)$$ (10)

where $X_{M_{ij}}$ is an element of the masked matrix, and $X_{ij}^{\prime }$ and *X*_*z*_ are elements from the shuffled and denoised matrices, respectively.

Importantly, the ZINB-based denoising autoencoder is trained on raw count data prior to masking, and masked entries are excluded from the ZINB likelihood. Masking is applied only to the denoised output for subsequent self-supervised representation learning.

**FIGURE 3:**
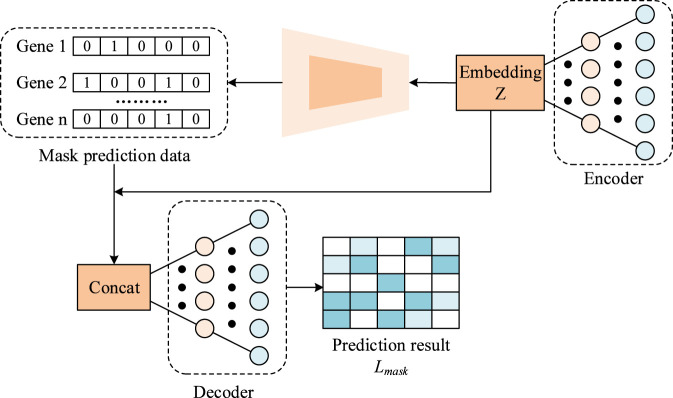
The structure of Mask Encoder-Decoder module.

Figure 3 shows the masking autoencoder, which consists of three main components: an encoder, a mask predictor, and a decoder. The encoder transforms the masked gene expression matrix *X*_*M*_ into a low-dimensional embedding *Z*. For an encoder with *F* layers, the output of the *f*th layer is computed as Equation 11.

$$Z_{f}=\sigma \left(W_{f}Z_{f-1}+b_{f}\right)$$ (11)

where σ is the activation function. The final layer applies a linear transformation (i.e., identity activation), and its output *Z*_*F*_ serves as the embedding *Z*.

To address potential inaccuracies in the masked input, the model first uses a mask predictor to estimate which expression values have been modified, producing a predicted mask matrix *M'*. It is implemented as a linear layer trained with cross-entropy loss as Equation 12.

$$L_{m}=-\sum_{ij}M_{ij}\log\left(M_{ij}^{\prime }\right)$$ (12)

The decoder reconstructs the gene expression matrix using the embedding Zand the predicted mask *M'*. A weighted mean squared error (MSE) loss is applied to emphasize masked genes as Equation 13. And weight *W_ij_* is defined as Equation 14.

$$L_{rec}=\frac{1}{N}\sum_{ij}W_{ij}\cdot {\left|X_{ij}-{\tilde{X}}_{ij}\right|}^{2}$$ (13)

$$W_{ij}=\lambda \cdot M_{ij}+\left(1-\lambda \right)\cdot \left(1-M_{ij}\right)$$ (14)

Here, *λ* is a hyperparameter assigning a higher weight to masked genes. The total loss is a weighted combination of the two objectives as Equation 15.

$$L_{mask}=\gamma_{m}\cdot L_{m}+\left(1-\gamma_{m}\right)\cdot L_{rec}$$ (15)

where *γ*_*m*_ balances the two terms. During clustering, only the embedding *Z* generated by the encoder is used.

To enhance the clustering performance of the model, a weighted soft clustering module is introduced. This module employs a weighted K-means approach to assign data points (cells) to cluster centers while preserving local similarity structures among cells with comparable gene expression profiles. The weighted K-means loss is defined as Equation 16.

$$L_{kmeans}=\sum_{k=1}^{n}\sum_{k=1}^{C}w_{ik}{\left\|z_{i}-c_{k}\right\|}^{2}$$ (16)

where *w*_*ik*_ is the weight for cell and cluster, *z*_*i*_ is the embedding of cell *i*, and *c*_*k*_ is the center of cluster *k*, updated by Equation 17.

$$c_{k}=\frac{\sum_{k=1}^{n}w_{ik}z_{ik}}{\sum_{k=1}^{n}w_{ik}}$$ (17)

The weight is computed using Equation 18.

$${\bar{w}}_{ik}=\frac{\exp\left(-{\left\|z_{i}-c_{k}\right\|}^{2}\right)}{\sum_{k=1}^{C}\exp\left(-{\left\|z_{i}-c_{k}\right\|}^{2}\right)}$$ (18)

Subsequently, the weights are sharpened using a Markov-like inflation step by Equation 19.

$$w_{ik}=\frac{{\bar{w}}_{ik}^{\alpha }}{\sum_{k=1}^{C}{\bar{w}}_{ik}^{\alpha }}$$ (19)

where is a hyperparameter (default 1). To better capture similarity relationships, a student’s t-distribution is used to model pairwise cell similarities. The soft assignment probability *q*_*ij*_ is given by Equation 20:

$$q_{ij}=\frac{{\left(1+{\left\|z_{j}-z_{i}\right\|}^{2}/t\right)}^{-\frac{t+1}{2}}}{\sum_{c\neq i}{\left(1+{\left\|z_{j}-z_{i}\right\|}^{2}/t\right)}^{-\frac{t+1}{2}}}$$ (20)

with *t* set to 1. A target distribution *p* is derived from *q* to strengthen high-confidence assignments as Equation 21.

$$p_{ij}=\frac{q_{ij}^{2}/\sum_{i\neq j}q_{ij}}{\sum_{c\neq i}\left(q_{ij}^{2}/\sum_{i\neq j}q_{ij}\right)}$$ (21)

The Equation 22 defines the clustering loss, the KL divergence between *p* and *q*:

$$L_{cluster}=\sum_{i}\sum_{j}p_{ij}\log\frac{p_{ij}}{q_{ij}}$$ (22)

The overall training objective combines all losses as Equation 23.

$$L=\alpha L_{zinb}+\beta L_{mask}+\varphi L_{kmeans}+\theta L_{cluster}$$ (23)

where *α*, *β*, *φ*, *θ* are tunable hyperparameters.

## 3 Experiment

This study evaluates the model’s clustering and classification performance using six annotated scRNA-seq datasets from both mouse and human. These datasets cover multiple biological systems, diverse cell types, and varying cluster sizes, and were generated using different RNA extraction protocols and sequencing platforms (e.g., Smart-seq, Drop-seq). The datasets—Adam, Deng, Muraro, Pollen, Chen, and Zeisel—are summarized in Table 1.

**TABLE 1:** Single-cell sequencing dataset used in the experiment.

Datasets	Cell count	Numbers of genes	Number of cell categories	Sequencing platform	Geo accession number
Adam	3,660	23,797	8	Drop-seq	GSE94333
Deng	268	22,431	6	Smart-seq	GSE45719
Muraro	2,126	19,127	10	CEL-Seq2	GSE85241
Pollen	301	23,730	11	SMARTer	GSE124299
Chen	12,089	23,284	46	Drop-seq	GSE87544
Zeisel	3,005	19,958	12	STRT-seq	GSE60361

For fair comparison, all baseline methods were implemented using recommended settings from their original publications or official repositories. Highly variable genes (HVGs) were selected using Scanpy with default parameters unless otherwise specified. Latent dimensionality was set to 10–32 depending on method defaults.

Graph-based methods (Seurat, graph-sc) used k-nearest neighbor graphs with k = 15–30 and Leiden clustering with default resolution. Deep clustering baselines were run with identical train/validation splits and optimized using Adam. For stochastic methods, each experiment was repeated multiple times with different random seeds, and the best-performing configuration was reported.

The base environment of the experimental platform utilizes an Intel Xeon E5-2,630 v4 CPU, a Tesla 2080Ti GPU with 24 GB of VRAM, and 64 GB of memory. The operating system is Ubuntu 18.04.6, PyTorch version 1.13.1, and Python 3.8.16.

### 3.1 Comparative clustering methods

This section compares the scDMAC method with PCA (Todorov et al., 2018) +K-means (Hartigan and Wong, 1979), Seurat (Tian et al., 2019), scDeepCluster (Eraslan et al., 2019), scziDesk (Chen et al., 2020), scVI (Lopez et al., 2018), graph-sc (Ciortan and Defrance, 2022), AutoClass (Li et al., 2022), scDCCA (Wang et al., 2023), CellBRF (Xu et al., 2023), and CTEC (Wang et al., 2024). Validation is performed across six public datasets, with results from some papers being reproducible.

PCA + K-means (Todorov et al., 2018; Hartigan and Wong, 1979) applies principal component analysis (PCA) to project high-dimensional scRNA-seq data into a lower-dimensional subspace, reducing noise and redundancy. K-means clustering is then performed in the reduced space to partition cells into groups by iteratively optimizing cluster centroids.

Seurat (Tian et al., 2019) first constructs a k-nearest neighbor graph based on gene expression profiles, then builds a shared nearest neighbor (SNN) graph to refine cell-to-cell similarities. Community detection is applied on the SNN graph to identify cell clusters.

scDeepCluster (Eraslan et al., 2019) employs a denoising autoencoder that injects Gaussian noise into the encoder to improve robustness. The model jointly learns a low-dimensional latent representation and cluster assignments using a KL divergence-based clustering loss, with a decoder utilizing a ZINB loss to model scRNA-seq data characteristics.

scziDesk (Chen et al., 2020) integrates a denoising autoencoder with a clustering module that alternates between data reconstruction and soft clustering. It applies a soft self-training K-means approach to iteratively refine cluster labels in the latent space.

scVI (Lopez et al., 2018) is based on a variational autoencoder (VAE) framework that uses a zero-inflated negative binomial (ZINB) likelihood to model scRNA-seq data, explicitly accounting for dropout events and over-dispersion. It infers a latent representation that is used for downstream clustering.

Graph-SC (Ciortan and Defrance, 2022) utilizes a graph autoencoder structure to model relationships between cells and genes. It can incorporate external biological networks (e.g., gene-gene interaction networks) to enhance the graph representation and improve clustering performance.

AutoClass (Li et al., 2022) adopts a dual-network architecture consisting of an autoencoder for denoising and feature extraction, and a classifier that promotes discriminative latent structures. The model is trained to preserve biological information while reducing technical noise.

scDCCA (Wang et al., 2023) applies deep canonical correlation analysis (DCCA) with a dual contrastive learning module to integrate multi-view information and learn invariant features. It aims to improve clustering by maximizing agreement between augmented views of the data.

CellBRF (Xu et al., 2023) introduces a random forest-based feature selection method within a spectral clustering pipeline. It employs a class-balancing strategy to mitigate the impact of imbalanced cell type distributions on gene importance estimation.

CTEC (Wang et al., 2024) is a cross-table ensemble clustering approach that combines multiple base clustering results using two refinement strategies: distribution-based and outlier-based reclustering, leading to a robust consensus partition.

These methods represent a range of classical and state-of-the-art approaches in scRNA-seq clustering, encompassing linear models, graph-based techniques, deep learning architectures, and ensemble strategies.

### 3.2 Evaluation metrics

In this study, Clustering performance is evaluated using external metrics that leverage known ground truth labels. This study employs the Adjusted Rand Index (ARI) and Normalized Mutual Information (NMI) for this purpose. The Rand Index (RI) measures similarity between the clustering result and true labels, defined as:

$$\text{RI}=\frac{TP+FN}{TP+TN+FP+FN}$$ (24)

where TP, TN, FP, and FN represent the numbers of true positives, true negatives, false positives, and false negatives in pairwise cluster assignments. To correct for chance agreement, the Adjusted Rand Index (ARI) is used:

$$\text{ARI}=\frac{RI-E\left(RI\right)}{\max\left(RI\right)-E\left(RI\right)}$$ (25)

ARI values near 0 indicate random clustering, while higher values reflect better alignment with true labels.

NMI assesses the mutual dependence between the clustering result *V* and true labels *U*, normalized by their entropies:

$$\text{NMI}\left(U,V\right)=\frac{2\cdot \text{MI}\left(U,V\right)}{H\left(U\right)+H\left(V\right)}$$ (26)

where MI(*U*, *V*)is the mutual information between *U* and *V*, and H(*U*), H(*V*) denote their entropies. NMI ranges between 0 (independent) and one (perfect match).

$$\text{MI}\left(U,V\right)=\sum_{u\in U}\sum_{v\in V}P\left(u,v\right)\log\left(\frac{p\left(u,v\right)}{p\left(u\right)p\left(v\right)}\right)$$ (27)

Where *p* (*u*,*v*) is the joint probability distribution function of *u* and *v*, *p*(*u*) denotes the probability of a data point belonging to the true class, *p*(*v*) denotes the probability of a data point belonging to the clustered class. Both ARI and NMI provide robust, normalized measures for comparing clustering performance across datasets.

### 3.3 Clustering results analysis

The ARI and NMI results of different methods across the datasets are summarized in Tables 2, 3, with the best and second-best performances highlighted in bold and underlined, respectively. We performed repeated runs with different random seeds and observed low variance across runs (typically <0.01 in ARI).

**TABLE 2:** ARI values for different methods on sequencing datasets.

Method	Adam	Deng	Muraro	Pollen	Chen	Zeisel
PCA + K-means	0.5354	0.6013	0.6810	0.7563	0.2284	0.4732
Seurat (Tian et al., 2019)	0.4973	0.3249	0.4463	0.7668	0.6213	0.3271
scDeepCluster (Eraslan et al., 2019)	0.8187	0.7294	0.7442	0.8832	0.3715	0.5164
scziDesks (Chen et al., 2020)	0.7894	0.8487	0.7973	0.8701	0.7902	0.6261
scVI (Lopez et al., 2018)	0.6197	0.3243	0.5017	0.8901	0.4796	0.3552
graph-sc (Ciortan and Defrance, 2022)	0.6217	0.8603	0.8042	0.8841	0.5361	0.6013
AutoClass (Li et al., 2022)	0.5321	0.8577	0.7654	0.8664	0.8527	0.5791
scDCCA (Wang et al., 2023)	0.9201	0.8794	0.8321	0.8967	0.8436	0.6257
CellBRF (Xu et al., 2023)	0.8193	0.8697	0.8127	0.7891	0.8211	0.6154
CTEC (Wang et al., 2024)	0.7087	0.8591	0.8191	0.8021	0.7993	0.6346
scCGC (Wang et al., 2023)	0.9311	0.8651	0.8541	0.9107	**0.8913**	0.7911
scDMAC (ours)	**0.9354**	**0.8896**	**0.8698**	**0.9151**	0.8821	**0.7983**

The best values highlighted in bold.

**TABLE 3:** NMI for different methods on sequencing datasets.

Method	Adam	Deng	Muraro	Pollen	Chen	Zeisel
PCA + K-means	0.6871	0.6140	0.6882	0.8663	0.6987	0.4732
Seurat (Tian et al., 2019)	0.7384	0.6595	0.7055	0.9011	0.7544	0.5817
scDeepCluster (Eraslan et al., 2019)	0.8401	0.7525	0.8110	0.8562	0.8237	0.6106
scziDesks (Chen et al., 2020)	0.8373	0.8657	0.8159	0.9022	0.7944	0.6368
scVI (Lopez et al., 2018)	0.7624	0.8545	0.8032	0.9231	0.7795	0.6242
graph-sc (Ciortan and Defrance, 2022)	0.7308	0.7473	0.7980	0.9304	0.7852	0.6417
AutoClass (Li et al., 2022)	0.6996	0.8631	0.7801	0.9283	0.7312	0.6391
scDCCA (Wang et al., 2023)	0.9021	0.8613	0.8207	**0.9431**	0.8476	0.7402
CellBRF (Xu et al., 2023)	0.8193	0.8794	0.8267	0.9197	0.8562	0.7297
CTEC (Wang et al., 2024)	0.7771	0.8752	0.8039	0.9064	0.7993	0.6915
scCGC (Wang et al., 2023)	0.9107	0.8691	**0.8541**	0.9427	0.8917	**0.7751**
scDMAC (ours)	**0.9224**	**0.8802**	0.8459	0.9363	**0.8973**	0.7692

The best values highlighted in bold.

As shown, scDMAC achieves the highest ARI values on five datasets (Adam, Deng, Muraro, Pollen, and Zeisel), demonstrating its robustness and accuracy in clustering cells from diverse platforms, tissues, and organisms. This result underscores the effectiveness of its masked autoencoder in feature learning and the advantage of weighted soft clustering. Compared to scCGC, scDMAC’s improved feature reconstruction loss leads to better noise suppression, particularly on the Deng and Muraro datasets, where the ARI improvement exceeds 0.01. On the Chen dataset, scDMAC’s ARI is less than 0.01 lower than the top method, which may be attributed to the presence of continuous or transitional cell types in this dataset, making clear cluster separation challenging.

In terms of average ranking based on ARI, scDMAC achieves the highest overall ranking, followed by scCGC, scDCCA, and CellBRF.

Regarding NMI, scDMAC also performs strongly, obtaining the highest scores on the Adam, Deng, and Chen datasets. On the Pollen dataset, scDCCA slightly outperforms scDMAC, likely due to its distributional regularization benefiting feature modeling on this data. Although scDMAC’s NMI is marginally lower than scCGC on the Muraro and Zeisel datasets, it remains competitive, affirming the overall efficacy of the proposed approach.

As shown in Table 4, scDMAC consistently achieves the highest silhouette scores on five out of six datasets (Adam, Deng, Muraro, Chen, and Zeisel), indicating superior intrinsic clustering quality compared to representative baseline methods. In particular, scDMAC shows clear improvements over PCA + K-means and scDeepCluster across all datasets, highlighting the benefit of deep representation learning combined with denoising and masking strategies.

**TABLE 4:** Silhouette score for different methods on sequencing datasets.

Method	Adam	Deng	Muraro	Pollen	Chen	Zeisel
PCA + K-means	0.4766	0.4204	0.3909	0.5663	0.4842	0.3606
scDeepCluster (Eraslan et al., 2019)	0.6001	0.4708	0.6110	0.5562	0.5585	0.4707
scDCCA (Wang et al., 2023)	0.6233	0.5701	0.7211	**0.6428**	0.5725	0.5438
scDMAC (ours)	**0.6477**	**0.5908**	**0.7374**	0.6230	**0.5942**	**0.5521**

The best values highlighted in bold.

Compared with scDCCA, scDMAC achieves comparable or higher silhouette scores on most datasets, with the only exception being the Pollen dataset, where scDCCA slightly outperforms scDMAC. This minor difference may be attributed to dataset-specific characteristics, such as a small number of cells and strong inter-gene correlations, which can favor contrastive learning–based representations. Overall, these results demonstrate that scDMAC produces more compact and well-separated clusters in the latent space, providing strong internal validation independent of external annotations.

**FIGURE 4:**
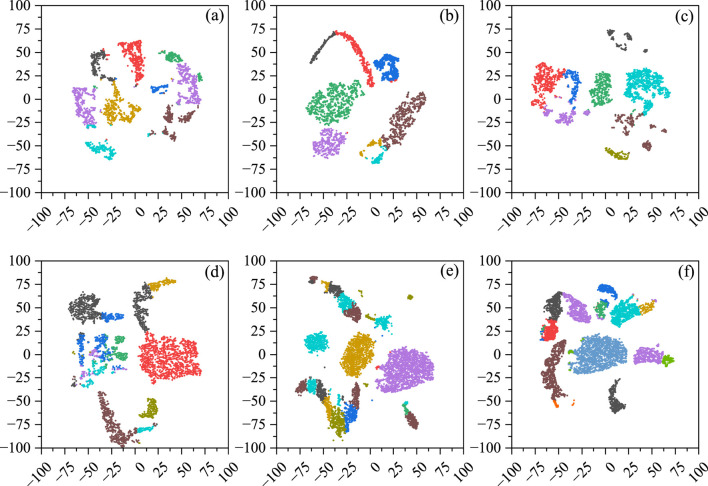
Visualization of scDMAC’s clustering results. **(a)** Adam, **(b)** Deng, **(c)** Muraro, **(d)** Pollen, **(e)** Chen, **(f)** Zeisel.

To validate the clustering performance, we applied t-SNE to visualize the six scRNA-seq datasets. As shown in Figure 4, the visualization results exhibit improved cluster separation and fewer outlier cells, indicating enhanced discrimination and denoising capability of the proposed model.

**FIGURE 5:**
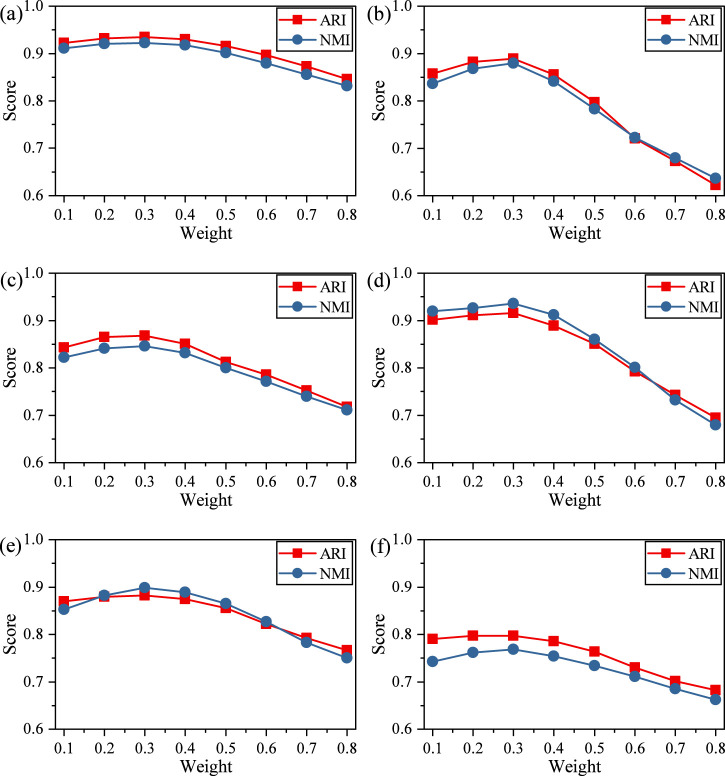
ARI and NMI values of scDMAC under different disturbance ratios. **(a)** Adam, **(b)** Deng, **(c)** Muraro, **(d)** Pollen, **(e)** Chen, **(f)** Zeisel.

We further evaluated the impact of the masking ratio on model performance. Figure 5 shows the ARI and NMI scores under masking ratios ranging from 0.1 to 0.8.

Performance improves as the ratio increases to 0.3, but declines with higher ratios, suggesting that excessive masking may hinder the model’s ability to reconstruct meaningful gene expression patterns. At low masking ratios (0.1–0.3), the model achieves peak or near-peak performance, with both ARI and NMI exhibiting their highest values in this range. For instance, ARI on the Adam, Deng, Muraro, and Pollen datasets reaches maxima around a masking ratio of 0.2–0.3, indicating that a moderate level of input perturbation effectively regularizes the model and enhances generalization. Performance at 0.2 and 0.3 is notably stable, with ARI improvements of ∼1–3% compared to 0.1 across most datasets.

When the masking ratio increases beyond 0.4, both ARI and NMI show a gradual decline, becoming substantial at masking ratios ≥0.6. This degradation suggests that excessive masking removes too much biological signal, making it difficult for the model to reconstruct informative representations needed for accurate clustering. The results indicate that a masking ratio in the range of 0.2–0.3 provides the optimal balance between regularization and information retention.

**FIGURE 6:**
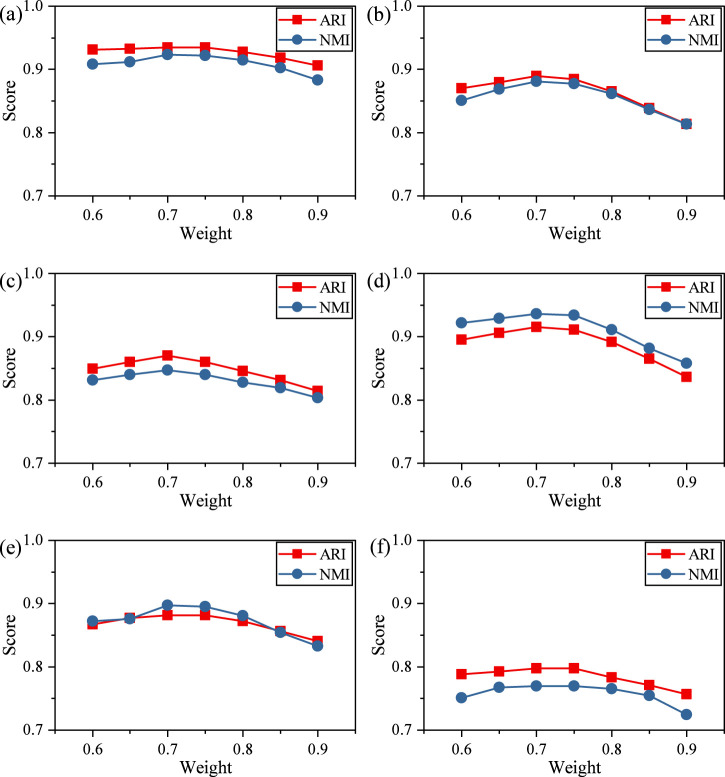
ARI and NMI values of scDMAC under Different Reconstruction Weights. **(a)** Adam, **(b)** Deng, **(c)** Muraro, **(d)** Pollen, **(e)** Chen, **(f)** Zeisel.

The weighting of the reconstruction loss also influences model behavior. Figure 6 illustrates the effect of varying the weight assigned to corrupted genes (from 0.6 to 0.9).

Across nearly all datasets, both ARI and NMI improve steadily from 0.6 to 0.7, reaching a performance peak around 0.7, where the model achieves its best or near-best scores, for example, Adam (ARI 0.93494, NMI 0.92289), Deng (ARI 0.88916, NMI 0.88072), and Pollen (ARI 0.91566, NMI 0.93614). Increasing the reconstruction loss beyond this range (≥0.8) leads to a noticeable and consistent decline in performance, suggesting that overly strong reconstruction constraints may hinder the model’s ability to learn discriminative low-dimensional features for clustering.

Importantly, the optimal region (0.65–0.75) is robust across datasets, indicating that the balance between reconstruction and clustering objectives is stable and not dataset-specific. Overall, these results demonstrate that selecting an appropriate reconstruction loss coefficient is critical, and a coefficient around 0.7 provides the best trade-off between representation fidelity and clustering separability.

**FIGURE 7:**
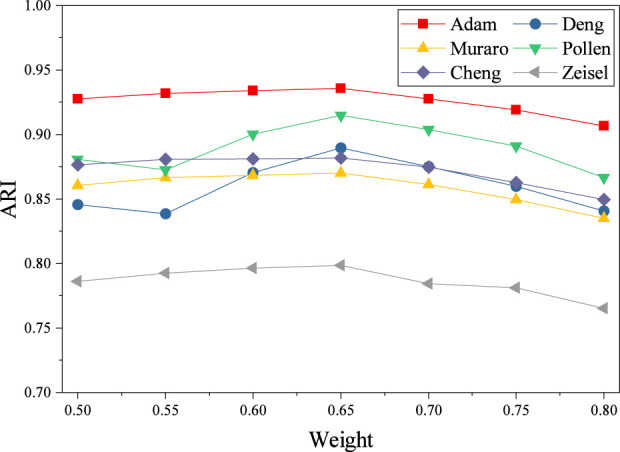
The curve of weight loss and ARI value. **(a)** Adam, **(b)** Deng, **(c)** Muraro, **(d)** Pollen, **(e)** Chen, **(f)** Zeisel.

Finally, the weight of the masking prediction loss was tuned between 0.5 and 0.8. As shown in Figure 7, the best overall performance is consistently observed at a masking-prediction loss of 0.65, which yields the highest or near-highest ARI values for all datasets, including Adam (0.935), Deng (0.89), Muraro (0.87), Pollen (0.91), Chen (0.88), and Zeisel (0.79). This suggests that a moderate masking-prediction strength provides an optimal balance between learning robust masked-feature representations and maintaining sufficient information for discriminative clustering. In contrast, further increasing the loss beyond 0.7 leads to monotonic degradation in clustering accuracy across datasets, indicating that excessive masking-prediction pressure may distort the learned latent space and weaken cluster separability. The consistent cross-dataset trend demonstrates that the optimal setting (approximately 0.65) is stable and generalizable.

### 3.4 Ablation experiments

To evaluate the contribution of each component in scDMAC, we designed three ablated variants: scDMAC-Z: without the denoising module; scDMAC-W: without the weighted reconstruction in the masking autoencoder; scDMAC-P: without the mask prediction module.

As shown in Table 5, removing the denoising module (scDMAC-Z) led to noticeable performance degradation, particularly on the Deng, Chen, and Zeisel datasets. For example, on Zeisel, ARI dropped from 0.7983 to 0.7694 and NMI from 0.7692 to 0.7413, indicating that denoising is essential for handling datasets with high technical noise. Removing weighted reconstruction (scDMAC-W) resulted in a moderate decline across most datasets. The most significant drop occurred on Pollen (ARI from 0.9151 to 0.9021), suggesting that the weighting mechanism helps capture key features in datasets with complex cell types. When the mask prediction module was ablated (scDMAC-P), performance decreased substantially, e.g., on Muraro, ARI fell from 0.8698 to 0.8426. This demonstrates the importance of adaptively estimating masked regions for robust feature learning. In summary, the full scDMAC model benefits from the synergistic effect of its denoising, weighted reconstruction, and mask prediction components, with each playing a distinct role in improving clustering accuracy and stability across diverse scRNA-seq datasets.

**TABLE 5:** Ablation experiments.

Method	Evaluation criteria	Adam	Deng	Muraro	Pollen	Chen	Zeisel
scDMAC-Z	ARI	0.9211	0.8412	0.8264	0.8973	0.8315	0.7694
	NMI	0.9102	0.8351	0.8127	0.9181	0.8434	0.7413
scDMAC-W	ARI	0.9268	0.8719	0.8507	0.9021	0.8643	0.7912
	NMI	0.9167	0.8658	0.8305	0.9217	0.8712	0.7624
scDMAC-P	ARI	0.9294	0.8543	0.8426	0.8918	0.8671	0.7881
	NMI	0.9183	0.8524	0.8231	0.9108	0.8781	0.7589
scDMAC	ARI	0.9354	0.8896	0.8698	0.9151	0.8821	0.7983
	NMI	0.9224	0.8802	0.8459	0.9363	0.8973	0.7692

## 4 Conclusion

This paper proposes scDMAC, a clustering model for single-cell RNA sequencing data that integrates a denoising autoencoder with a masking autoencoder. The model first denoises scRNA-seq data using a ZINB-based denoising autoencoder to better approximate the underlying expression distribution. It then introduces variability by randomly shuffling expression values within genes and applies a Bernoulli-based masking strategy to generate perturbed gene expression profiles. These are encoded into low-dimensional embeddings through a masking autoencoder, which jointly optimizes feature reconstruction and mask prediction. Finally, a weighted soft clustering mechanism is applied to produce the clustering results.

Experimental results demonstrate that scDMAC achieves improved performance by effectively capturing gene-wise relationships and enhancing feature robustness. While simulation studies provide controlled ground truth, they often fail to capture the complex noise structure and biological heterogeneity of real scRNA-seq data. In this work, we prioritize evaluation on well-annotated benchmark datasets that are widely used in literature. Nevertheless, we acknowledge this limitation and plan to include simulation-based validation in future work.

## Data Availability

The original contributions presented in the study are included in the article/supplementary material, further inquiries can be directed to the corresponding author.
